# Maya Vanilla (*Vanilla cribbiana* Soto Arenas): A New Species in Commerce

**DOI:** 10.3390/plants14030300

**Published:** 2025-01-21

**Authors:** Araceli Pérez-Silva, Eduardo Peña-Mojica, Abimael Ortega-Galeana, Jocelyn I. López-Cruz, Carlos A. Ledesma-Escobar, Mónica Rivera-Rivera, Ernestina Paz-Gamboa

**Affiliations:** 1 TecNM/Instituto Tecnológico de Tuxtepec, Departamento de Ingeniería Química y Bioquímica, Tuxtepec 68350, Oaxaca, Mexico; epmojica09@hotmail.com (E.P.-M.); ibq.17350047@gmail.com (A.O.-G.); jocelynlopez714@gmail.com (J.I.L.-C.); monica.rr@tuxtepec.tecnm.mx (M.R.-R.); ernestina.pg@tuxtepec.tecnm.mx (E.P.-G.); 2 Department of Analytical Chemistry, University of Córdoba, 14071 Córdoba, Spain; z32leesc@uco.es; 3 Chemical Institute for Energy and Environment (iQUEMA), University of Córdoba, 14071 Córdoba, Spain

**Keywords:** *Vanilla cribbiana*, Maya vanilla, aroma compounds, ethnobotanic, food flavoring

## Abstract

Vanilla-producing regions in Mexico and around the world are experiencing declining yields due to global climate change. However, Mexico, Guatemala, and other parts of Central America possess underutilized genetic resources within the *Vanilla* genus, which can be used to increase their production. One such resource is *Vanilla cribbiana* Soto Arenas, known as Maya vanilla, which is native to Guatemala and Mexico. This study evaluated some of the physical and chemical characteristics as well as the aromatic and fatty acid profiles of cured vanilla pods of Maya vanilla. A 5 kg batch of cured vanilla pods from Cobán, Guatemala, was analyzed for length, weight, humidity content, and proximate chemical composition and aromatic profile using HPLC-DAD and GC-MS. The pod lengths ranged from 6 to 16 cm, and weights ranged from 2.2 to 8.2 g. The humidity content varied between 22% and 38.63%. The main component in the cured vanilla pods was insoluble crude fiber (51.18%). The vanillin, vanillic acid, *p*-hydroxybenzoic acid, and *p*-hydroxybenzaldehyde concentrations in the cured vanilla beans were 2.13 ± 0.68, 0.105 ± 0.035, 0.38 ± 0.05, and 0.345 ± 0.115 g/100 g dry matter, respectively. A total of 70 volatile compounds were identified with GC-MS—16 acids, 12 alcohols, 8 aldehydes, 15 esters, 12 hydrocarbons, 5 ketones, and 2 furans—among which were compounds characteristic of other commercial vanilla species. Oleic acid and linoleic acid represented over 82% of the total fatty acids. This study provides fundamental insights into the physicochemical and aromatic characteristics of Maya vanilla, highlighting the differences between this species and vanilla species traditionally used in commerce. *Vanilla cribbiana* Soto Arenas represents an excellent alternative for the vanilla market as a flavoring agent for the food and perfume industries.

## 1. Introduction

The “vanilla aroma”, represented by vanillin, is considered the most popular aroma worldwide [1]. However, less than 1% of vanillin originates from the fruits of orchids in the *Vanilla* genus [2]. *V. planifolia* Jacks. ex Andrews is the most commercially significant species, followed by *V. × tahitensis* J.W. Moore [3,4,5]. The high market prices of vanilla are due to the labor-intensive cultivation and curing processes [2,6]. Additionally, production is severely affected by deforestation, pests, diseases, and climate change [4,5,7,8,9]. Most of the demand for the vanilla aroma is met with more economical alternatives, such as chemically synthesized vanillin, which exhibits significant differences compared to the natural vanilla aroma [4,10,11,12,13].

In Mexico, vanilla has been known since pre-Hispanic times and was first depicted in the Codex de la Cruz-Badianus [14,15]. The Mexica called it *Tlixóchitl*, while the Totonacs referred to it as *Xhanat*, and the Maya as *Sisbic* or *Zizbic* [15,16,17,18,19]. Since then, vanilla has been a valuable commodity, used as currency like cacao. In the past, its main uses were medicinal, religious (for perfuming temples), in chocolate preparation, and as a tribute [18]. Aztec-descended emperors received vanilla from conquered peoples as a symbol of their greatness and power [14,17,20]. In Mexico, vanilla holds biocultural and economic value, particularly in the Totonacapan region [21]. Cured vanilla beans of the *V. planifolia* species have been used as ointments, in herbal teas to soothe coughs, as a stimulant, energizer, aphrodisiac, and for other purposes [15,16,20,22]. Today, although to a lesser extent, the medicinal uses of vanilla persist among the residents of southern Maya lands. They also use other parts of the plant, such as leaves, stems, and flowers, to alleviate headaches, abdominal pain, and depression; to heal wounds, vomiting, and ulcers; and to accelerate childbirth [19]. Scientific studies have confirmed that vanillin and its synthetic analogs regulate gene expression and exhibit biological activities, such as antioxidant, anticancer, antimicrobial, neuroprotective, and antimutagenic effects, among others. The authors of [23,24,25,26] developed interesting studies aimed at understanding the action of certain compounds, including vanillin, on ferroptosis and apoptosis in individual live cancer cells using an integrated transcriptome system.

Since pre-Hispanic times, the Maya peoples of Mexico and Guatemala have used wild-growing vanilla in regions such as the Lacandon Jungle (Mexico), the Petén, and Alta Verapaz (Guatemala). Among the vanilla species locally consumed since pre-Columbian times is a thick, triangular, aromatic vanilla variety [18]. This vanilla is ground on a *metate* along with cacao and annatto to form a chocolate paste. This combination of cacao, annatto, and vanilla is considered the cultural triad of the Maya in the southern lowlands, cultivated in agroforestry systems characteristic of the Maya [18,27]. The Maya’s agricultural success stemmed from their use of their biodiversity-rich environment, particularly plants used for food and medicine [28]. It is now known that the vanilla species used in the Maya cultural triad is *V. cribbiana* Soto Arenas [18,29].

**Figure 1:**
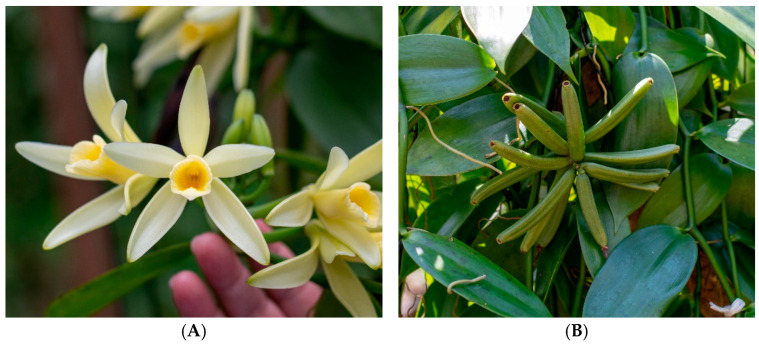
*Vanilla cribbiana* Soto Arenas (Maya vanilla). (**A**) Flower. (**B**) Fruits from “Rápidos Che’ sib’ ik” Farm, Coban, Alta Verapaz, Guatemala. Photographs by Araceli Pérez-Silva (2022).

*V. cribbiana* Soto Arenas is one of the 38 aromatic vanilla species reported in the Neotropical region [30]. This species belongs to the *Vanilla hostmanii* group and features large, white flowers with yellow-orange lips and aromatic fruits (Figure 1) [29,31]. *V. cribbiana* Soto Arenas has been documented in Mexico, Guatemala, Belize, Honduras, Colombia, Peru, and Brazil [21,29,32,33]. Although threatened by land-use changes and climate change [8,21], this species has been domesticated. Currently, there are plantations in the Alta Verapaz region of Guatemala where it is known as Maya vanilla. However, there is no published information on the physicochemical and aromatic characteristics of this species in commerce. Investigating other vanilla species beyond the commercially recognized ones is an urgent task to increase production and harness the biodiversity of the *Vanilla* genus in regions where they are endemic. Additionally, it diversifies vanilla aromatic profiles in commerce [4,5,9,32]. The use of plant genetic resources in crop improvement is an alternative for conserving valuable genetic resources while increasing agricultural production and food security [34]. In this regard, the utilization of germplasm from the *Vanilla* genus is an alternative to increase vanilla production and, simultaneously, an opportunity to improve the socioeconomic conditions of the inhabitants of areas where Maya vanilla is endemic, such as southern Mexico and Guatemala, regions that face serious migration challenges. However, this will only be possible if the aromatic attributes of this species, wild species of vanilla or developed vanilla hybrids that have adapted to climate change, are comparable to those of *V. planifolia*, the most important species in commerce [4]. This is because the economic value of vanilla is primarily determined by the vanillin and moisture content and pod size [35,36,37,38].

The importance of this study lies in generating, for the first time, scientific information on the physical characteristics, proximate chemical composition, semi-volatile compounds with HPLC-DAD, volatile compounds with GC-MS, and the fatty acid profile of cured Maya vanilla pods. The results provide insight into why this species was domesticated, the ethnobotanical value attributed to it by Maya culture, and its value as a new alternative in the vanilla market.

## 2. Results

The main quality attributes of vanilla have been primarily described for the species *V. planifolia* and are established in the ISO 5565-1:1999 [35], NMX-FF-074-SCFI-2009 [36], and NOM-182-SFCI-2011 [37] standards. In these standards, vanilla is primarily classified based on its physical attributes (moisture content and size) and vanillin content. The results obtained from the characterization of Maya vanilla regarding these attributes will be analyzed considering these standards, as well as information from studies reported in the literature on commercial species, *V. planifolia* from different origins, the *V. × tahitensis* hybrid, and, to a lesser extent, *V. pompona*. This is to enable a comparison of the physicochemical and aromatic characteristics of cured *V. cribbiana* Soto Arenas pods with those of the vanilla from commercial species.

### 2.1. Physical Characteristics of Cured Beans

**Figure 2:**
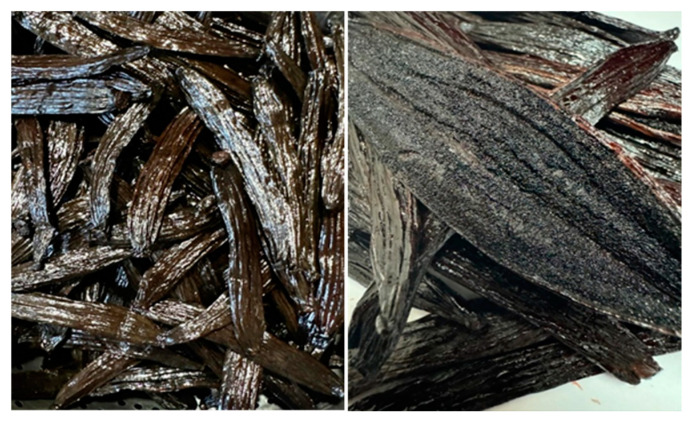
Cured vanilla beans of *V. cribbiana* Soto Arenas (Maya vanilla). Photographs by Araceli Pérez-Silva (2022).

The physical characteristics of the cured Maya vanilla pods (*Vanilla cribbiana* Soto Arenas) are shown in Table 1. Maya vanilla pods are very shiny and have abundant seeds (Figure 2). The longest pods measured 16 cm, corresponding to Category II beans of *V. planifolia* according to NMX-FF-074-SCFI-2009 [36]. However, the weight of some analyzed pods (>8 g) exceeded the weight of the cured *V. planifolia* beans classified as extra gourmet quality (5 g), considering that 1 kg of gourmet vanilla typically contains about 200 pods.

The humidity content of the pods is one of the most important parameters for measuring vanilla quality. The humidity content of the cured *Vanilla cribbiana* pods ranged between 22% and 38.63%. This range aligns with the values established for *V. planifolia* pods in different categories as per the ISO 5565-1:1999 [35], NMX-FF-074-SCFI-2009 [36], and NOM-182-SFCI-2011 [37] standards. This last regulation corresponds to the appellation of origin standard for Papantla Vanilla. The cured Maya vanilla pods with the highest moisture content (38%) are considered the highest quality pods (Category I) according to the ISO 5565-1:1999 [35] and NOM-182-SFCI-2011 [37] standards, whereas the pods with the lowest moisture content (22%) correspond to ordinary Category II according to the NMX-FF-074-SCFI-2009 standard. In this standard, lower-quality pods are specified to have a moisture content range of 10 to 20% and are classified as ordinary Category III for *V. planifolia* pods.

### 2.2. Proximate Chemical Composition

The results of the proximate chemical composition analysis are described in Table 2. Fiber was the predominant component (50.19%), composed of 26.42% lignin, 12.71% hemicellulose, and 11.06% cellulose. Fats were the second most significant component (13.96%), followed by ash (7.30%). Finally, carbohydrates were determined according to the difference, accounting for 23.48%.

### 2.3. Analysis of Aromatic Compounds with HPLC-DAD

**Figure 3:**
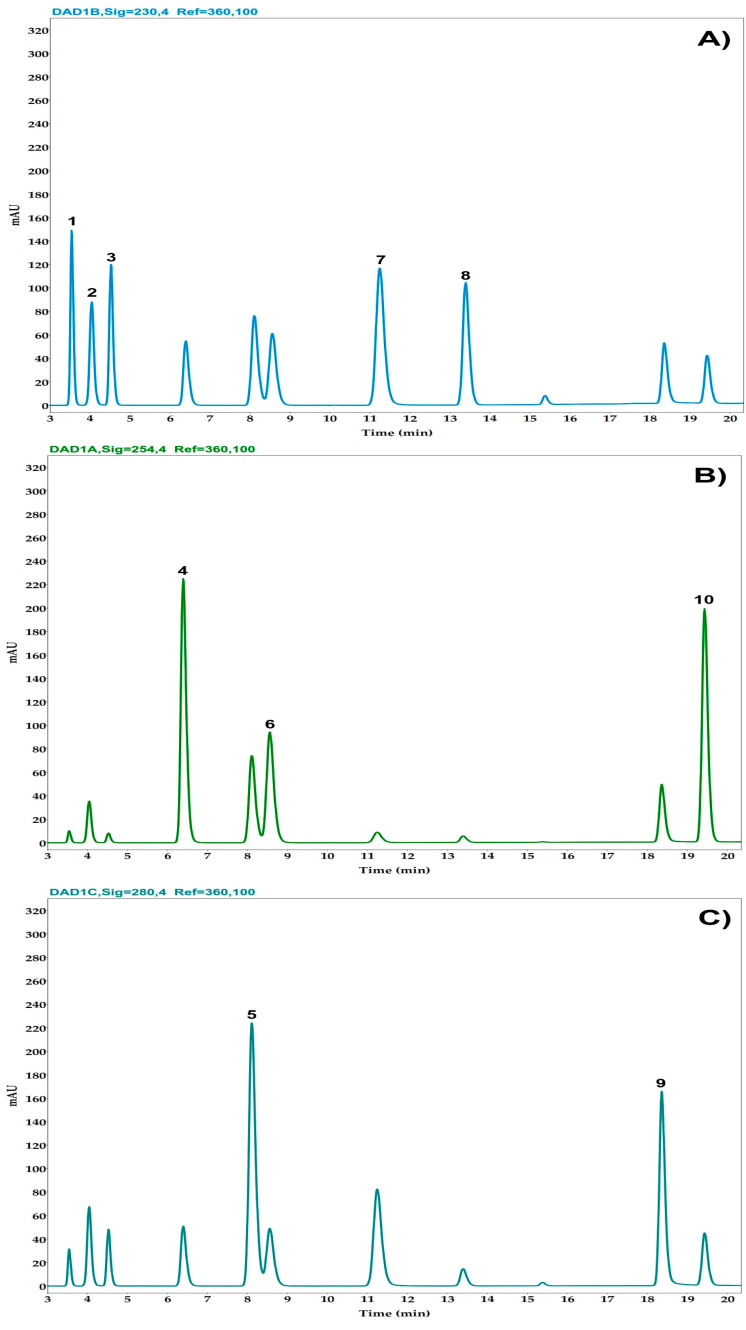
HPLC-DAD chromatogram at 230 nm (**A**), 254 nm (**B**), and 280 nm (**C**) of standard compounds: *p*-hydroxybenzyl alcohol (1), glucovanillin (2), vanillyl alcohol (3), *p*-hydroxybenzoic acid (4), *p*-hydroxybenzaldehyde (5), vanillic acid (6), vanillin (7), anisyl alcohol (8), anisaldehyde (9), and anisic acid (10).

**Figure 4:**
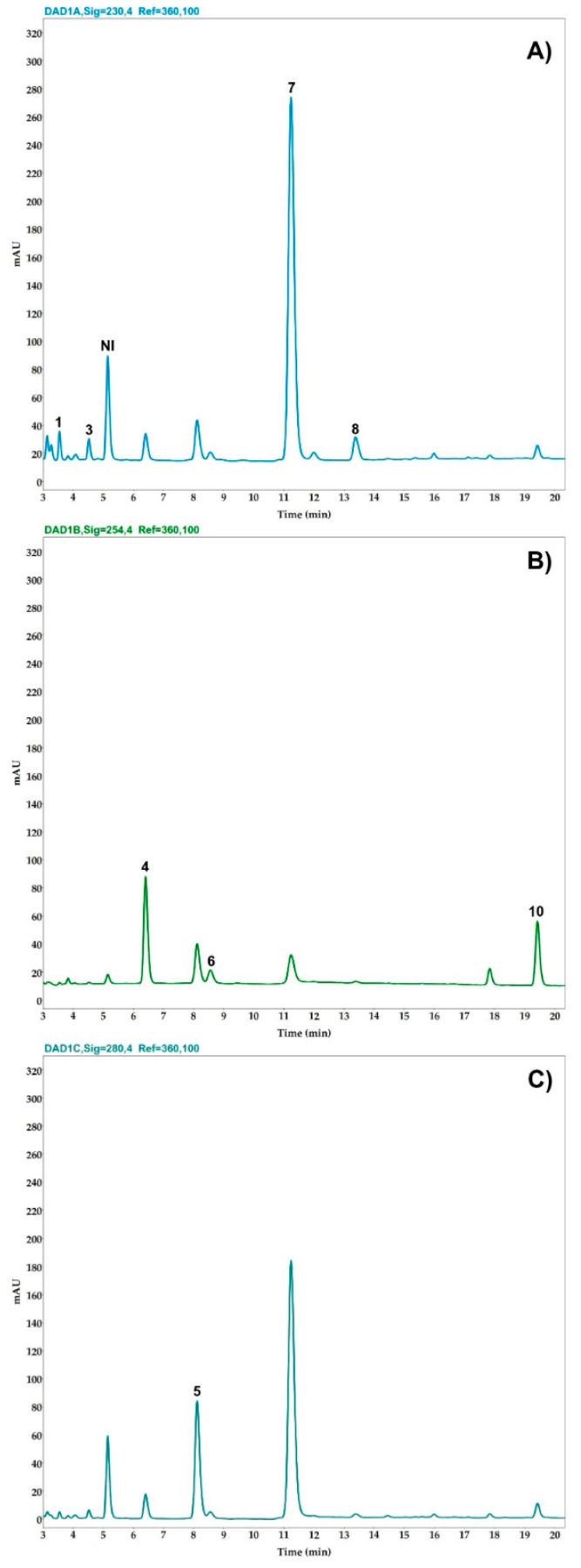
HPLC-DAD chromatogram at 230 nm (**A**), 254 nm (**B**), and 280 nm (**C**) of cured *Vanilla cribbiana* Soto Arenas beans: *p*-hydroxybenzyl alcohol (1), vanillyl alcohol (3), *p*-hydroxybenzoic acid (4), *p*-hydroxybenzaldehyde (5), vanillic acid (6), vanillin (7), anisyl alcohol (8), and anisic acid (10). NI: Not identified.

The analysis quantified ten compounds: glucovanillin, vanillin, vanillic acid, vanillyl alcohol, *p*-hydroxybenzaldehyde (*pHB*), *p*-hydroxybenzoic acid (*pHB acid*), *p*-hydroxybenzyl alcohol (*pHB alcohol*), anisaldehyde, anisyl alcohol, and anisic acid (Figure 3). In Figure 4, the chromatogram of the analysis of the cured *Vanilla cribbiana* beans is shown. Details of these are provided in Table 3. The values set by the NOM-182-SFCI-2011 [37] standard for vanilla from Papantla, corresponding to *V. planifolia*, are also presented. It can be observed that the values of the aromatic quality indicator compounds established for *V. planifolia* are lower than those determined in Maya vanilla (Table 3). The vanillin concentration in the cured vanilla beans of *V. cribbiana* was 2.13 ± 0.68 g/100 g dry matter. In addition to the presence of the compounds characteristic of *V. planifolia, V. cribbiana* contains anisyl compounds that are characteristic of *V. × tahitensis* and *V. pompona* [11,39]. Glucovanillin was not detected in the cured Maya vanilla beans, indicating that the glucosylated precursor was fully hydrolyzed during the vanilla curing process. Anisaldehyde could not be quantified in the samples analyzed using HPLC-DAD due to its low concentration, so this compound was identified using GC-MS.

### 2.4. Aromatic Profile Analysis with GC-MS

The analysis of organic extracts using GC-MS identified 70 volatile compounds (Table 4). The compounds identified in Maya vanilla were categorized into seven families: acids (16), alcohols (12), aldehydes (8), esters (15), furans (2), hydrocarbons (12), and ketones (5).

### 2.5. Fatty Acid Profile Analysis with GC-MS

The fatty acid profile of the cured vanilla beans, as determined with GC-MS, is presented in Table 5. The results indicate that the fatty acid composition of Maya vanilla consists of saturated (16.99%), monounsaturated (53.61%), and polyunsaturated (29.41%) fatty acids. Oleic acid was the predominant fatty acid (~53%), followed by linoleic and palmitic acids.

## 3. Discussion

### 3.1. Physical Characteristics of Cured Pods

The size of *V. cribbiana* Soto Arenas pods is small compared to commercial vanilla species. However, in the food industry, vanilla is predominantly marketed as a hydroalcoholic extract [22,40,41]. The pods are typically cut for these extracts, with aromatic quality being the primary attribute of interest. Therefore, the smaller size of *V. cribbiana* compared to other species does not pose an issue for its commercialization. Additionally, the thickness of the larger cured vanilla beans allows them to be easily opened longitudinally, facilitating the extraction of their abundant pulp and seeds for direct use. The weight of the pods depends on their humidity and length; the larger pods with humidity levels exceeding 35% were the heaviest. The humidity levels reported in cured *V. planifolia* pods from various origins range between 12% and 38% [35,36,37,42]. Specifically, the designation of origin for Papantla vanilla establishes humidity values between 24% and 38% for cured *V. planifolia* pods [37]. In contrast, Tahitian vanilla has reported humidity levels ranging from 44% to 50% [43,44]. Thus, the humidity range of Maya vanilla falls within the values reported for cured *V. planifolia* pods.

### 3.2. Proximate Chemical Composition

There are few studies on the proximate chemical composition of cured vanilla pods, with most focusing on the mature fruits or cured pods of *V. planifolia*. Peña-Barrientos et al. [45] reported that vanilla bean waste, after the alcohol extraction of aromatic compounds, retained 48.6% insoluble fiber, preserving 97% of the lignocellulosic compounds present in the raw material. This value is close to the 50.19% fiber content quantified in the Maya vanilla pods. The lipid content in Maya vanilla is similar to the maximum value determined in *V. madagascariensis* [46]. The ash and protein contents of Maya vanilla also align closely with that reported for vanilla bean waste [45]. The protein content quantified in Maya vanilla falls within the range reported for cured *V. planifolia* pods [47]. However, while the protein content in the mature fruits of *V. planifolia* may be higher, it tends to decrease in the cured pods due to senescence and a series of enzymatic reactions triggered during cellular decompartmentalization. These reactions promote the interaction of substrates with enzymes such as proteases, glucosidases, peroxidases, and polyphenol oxidases [42,48]. Heat or freezing during the “killing” stage and the activity of proteases are the primary factors responsible for protein denaturation. Carbohydrates in cured vanilla beans include organic acids, pectins, gums, mucilages, and sugars (sucrose, fructose, and glucose) [49]. The glucose released after the hydrolysis of glycosylated precursors and other simple sugars may decrease during the curing process as these dissolve in the water lost during pod dehydration.

The mineral content, as well as other components, depends significantly on agronomic practices. Studies have shown that the macronutrient and micronutrient content in vanilla plants is strongly influenced by the type of substrate used for cultivation [50].

### 3.3. Aromatic Compounds Quantified with HPLC-DAD

The aromatic compounds of vanilla quantified using HPLC-DAD have a glycosylated origin [4,51,52]. These aromatic precursors derive from the shikimic acid pathway [51] and are stored in the cells in a structure called phenyloplates [53]. The proportion of these glycosylated aromatic precursors in vanilla primarily depends on its origin, species (*V. planifolia* or *V. pompona*), or natural hybrid (*V. × tahitensis*), or those generated by interspecific hybridization (*V. planifolia* with *V. × tahitensis)* [4]. For vanilla to develop its aroma, the vanilla pods must undergo the curing process, during which glycosylated precursors are hydrolyzed by the action of the β-glucosidases present in the fruit [51], allowing the released aglycones to be detectable by the olfactory system. Glucovanillin is the main aromatic precursor in the ripe pods of *V. planifolia*. In cured vanilla beans, its presence is almost negligible. The concentration of vanillin, a product of the hydrolysis of glucovanillin, is the primary quality criterion in *V. planifolia* [35,36,42,44]. In *V. planifolia*, vanillin accounts for 80–85% of the total aromatic compounds, while in *V. × tahitensis*, it constitutes less than 50% [3,10,43]. Although it is not the only quality attribute, its economic value is directly related to the concentration of this compound. Vanilla is the second most expensive species after saffron. Between 2017 and 2018, the price of vanilla increased to USD 600 per kilogram [54]. Vanilla has become as expensive as silver [55]. Although the price of vanilla decreased after the COVID-19 pandemic, it remains an expensive commodity. In this study, the average vanillin content determined in Maya vanilla is close to the maximum reported in *V. planifolia* from different origins and categories [3,4,10,37,44,56]. The vanillin content (2.13 ± 0.68%) identified in Maya vanilla is higher than that reported in *V. × tahitensis* (1.4–2.1%) [3]. For the other three major compounds in *V. planifolia*, the vanillic acid content (0.105 ± 0.035%) in Maya vanilla was similar to *V. planifolia*, while *p*-hydroxybenzaldehyde (0.345 ± 0.115%) and *p*-hydroxybenzoic acid (0.33 ± 0.05%) contents were higher [10,52]. In Maya vanilla, the anisyl compounds quantified with HPLC-DAD corresponded to anisyl alcohol and anisic acid, which are characteristic of *V. pompona* and *V. × tahitensis* [3,43,44,57,58]. In Tahitian vanilla, anisyl compounds represent 45% of the aromatic compounds [43]. The presence of anisyl compounds in *V. cribbiana* is advantageous as they contribute to anise/spicy and floral notes, which are key markers in *V. × tahitensis* [43,57].

### 3.4. Aromatic Profile According to GC-MS

The aroma of vanilla is complex and sophisticated [10,11,12,58], resulting from a combination of non-volatile, semi-volatile, and volatile compounds. Volatile compounds are regarded by specialists as aroma fortifiers or synergists in vanilla’s characteristic scent [58]. The aromatic profile of vanilla pods is influenced by the species, production location, curing process, and storage conditions [4,58]. In Maya vanilla, compounds with diverse aromatic attributes were identified, including descriptors such as vanilla, woody, balsamic, anise, sweet, herbal, fruity, floral, smoky, phenolic, wine-like, and others. These descriptors have been reported in the analyses of aroma-active compounds conducted using GC-O and the sensory evaluations of *V. planifolia* and *V. × tahitensis* pods [10,11,12,43,57,59]. The aromatic notes of compounds largely depend on their chemical structure and functional groups. For instance, in the *Vanilla* genus, anisic compounds are primarily responsible for anise, fruity, floral, and sweet notes, characteristic of *Vanilla × tahitensis* and *V. pompona* [11,39,43,57]. These notes are mainly attributed to the presence of anisyl alcohol, anisaldehyde, anisic acid, and methyl *p*-anisate. This family of compounds is also responsible for the floral notes in *V. cribbiana* Soto Arenas [60]. Vanillyl compounds, including vanillin, vanillic acid, dehydrozingerone, and others, are responsible for vanilla, balsamic, and spicy notes. This group of compounds is predominant in *V. planifolia* [10,13,43,52]. Benzyl compounds, such as benzyl alcohol, *p*-hydroxybenzyl alcohol, benzyl acetate, and benzoic acid, generate descriptors for floral, fruity, and balsamic notes. These compounds have also been reported in commercial vanilla species [44,58]. Lastly, phenolic compounds, including phenol, guaiacol, *p*-cresol, *p*-creosol, and *p*-vinylphenol, produce phenolic, medicinal, chemical, and smoky notes. This family is consistently present in the three major commercial vanilla species (*V. planifolia*, *V. × tahitensis*, and *V. pompona*) [10,11]. High concentrations of these compounds in pods can impart unpleasant odors. While their presence cannot be avoided, they can be managed during curing, particularly guaiacol and creosol [59,61]. In *V. cribbiana*, 15 alcohols were identified, including the derivatives of shikimic acid, linear alcohols, and terpenes. The aromatic descriptors of these compounds are diverse, including floral, waxy, and citrusy. This group also includes two terpenes, borneol and fenchol, which are responsible for camphor, pine, and woody notes. Methyl *p*-anisate has been reported as an aroma-active compound in *V. × tahitensis* [11], and methyl cinnamate in *V. planifolia* [10,12]. Both esters were identified in Maya vanilla. Regarding the acids identified in *V. cribbiana*, the profile includes both short- and long-chain fatty acids, as well as acetic acid, which has been identified as an aroma-active compound in *V. planifolia* [10,59].

### 3.5. Fatty Acid Profile Analysis

To date, the only study on the fatty acid content in *V. planifolia* and *V. × tahitensis* was conducted by Brunschwig et al. [3], who reported significant differences in fatty acid content between the two species. *V. × tahitensis* has a higher fatty acid content, which accounts for the oily appearance of its pods. This oily characteristic is also observed in the pods of *V. cribbiana* Soto Arenas (Figure 2), where oleic acid is the most abundant fatty acid (~53%), followed by linoleic acid (29.41%). This order is reversed in *V. × tahitensis* [3]. Some aldehydes present in vanilla are the result of the autoxidation of oleic and linoleic acids, explaining their decrease during the curing process [43,52].

Liu et al. [62] reported that unsaturated fatty acids offer significant therapeutic potential in preventing oxidative stress and inflammation, as well as cardiovascular and cerebrovascular diseases, cancer, and osteoporosis. Therefore, the content of unsaturated fatty acids in Maya vanilla may contribute to its bioactive properties, which should be studied in greater depth.

This study is an initial approach to the characterization of Maya vanilla. It was conducted through routine analyses using the equipment established in the standards for determining the main quality parameters of vanilla. Recent reports have indicated using the methodology to rapidly authenticate the purity of vanilla extracts (*V. planifolia*) by applying a multivariate approach using near-infrared (NIR), mid-infrared (MIR), and Raman spectroscopy [63]. However, to further investigate the compounds responsible for the aromatic and bioactive qualities present in vanilla from different species, the application of emerging technologies, such as surface-enhanced Raman spectroscopy (SERS), which is highly sensitive in detecting very low concentrations of analytes, represents an excellent alternative method for evaluating the quality of vanilla [64].

## 4. Materials and Methods

### 4.1. Raw Materials

**Figure 5:**
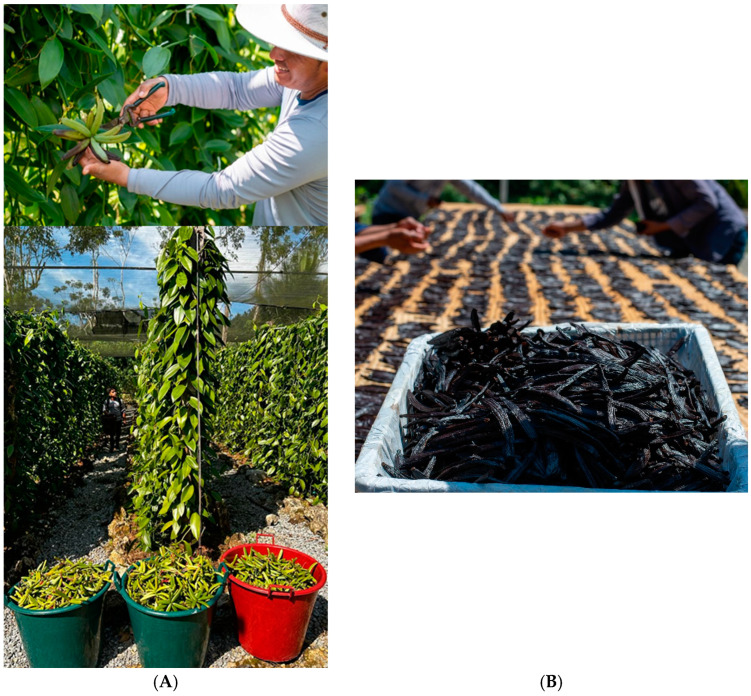
Harvest of fruits (**A**) and curing of *V. cribbiana* Soto Arenas beans (Maya vanilla) (**B**) from “Rápidos Che’ sib’ ik” Farm, Coban, Alta Verapaz, Guatemala. Photographs by Darío Ramírez-Fontana (2022).

Cured Maya vanilla beans (*Vanilla cribbiana* Soto Arenas) were obtained from a plantation spanning 3 hectares at the “Rápidos Che’ sib’ ik”, Coban, Alta Verapaz, Guatemala. The fruits were harvested in 2022 and cured at least nine months after flower pollination (Figure 5A). The traditional curing of the vanilla was carried out by vanilla producer Mr. Dario Ramirez Fontana. The traditional curing process involved killing the fruits in hot water at 80 °C for 10 s, followed by sweating (24 h). This sweating was alternated with sun-drying (2 to 4 h per day), and the process was repeated 20 to 25 times, depending on the size of the beans, over a period of 2 months. Finally, the vanilla beans were placed in a wooden box for one month for conditioning. The traditional curing of the Maya vanilla lasted 3 months. A batch of 5 kg of cured Maya vanilla, vacuum-packed, was sent to the laboratory of the TecNM/Instituto Tecnológico de Tuxtepec for analysis (Figure 5B).

### 4.2. Reagents

Glucovanillin was obtained from Chromadex (Irvine, CA, USA), and vanillin, vanillic acid, *p*-hydroxybenzaldehyde, *p*-hydroxybenzoic acid, vanillyl alcohol, *p*-hydroxybenzyl alcohol, anisaldehyde, and anisyl alcohol of analytical grade were obtained from Sigma-Aldrich (St. Louis, MO, USA). A mix of alkanes C8–C20 from Sigma-Aldrich (St. Louis, MO, USA) was used for identification with GC-MS.

### 4.3. Physical Analysis of Cured Vanilla Pods

The length was determined using a measuring tape, and the weight was determined using an OHAUS analytical balance model AX224 (Parsippany, NJ, USA). The measurements were taken from one hundred pods.

### 4.4. Determination of Moisture

The beans (2–3 g) were subjected to drying in an oven at 105 °C until a constant weight was obtained (approx. 24 h) [10,52]. One hundred pods of different sizes were analyzed after a physical analysis.

### 4.5. Proximate Chemical Analysis

The determination of fat, protein, and ash content was carried out according to the Association of Official Analytical Chemists [65], and the fiber content was determined according to Van Soest [66]. The carbohydrates can be calculated using the difference obtained by subtracting the amount of protein, fat, ash, moisture, and fiber from the total sample weight. The analyses were performed in triplicate using a mixture of pods of varying sizes.

### 4.6. Analysis of Volatile Compounds

#### 4.6.1. HPLC-DAD Analysis

The volatile compounds in the vanilla beans were quantified with HPLC-DAD according to the protocol established by Pérez-Silva et al. [10,52]. Briefly, 300 mg vanilla pod powder was added to 10 mL of methanol/H_3_PO_4_ 10^−2^ M (30/80; *v*/*v*) mixture; then, the resulting mixture was sonicated in an ultrasonic bath at a 37 kHz frequency for 10 min at room temperature. The extract was filtered using a paper filter and passed through a 0.45 µm filter prior to the HPLC-DAD analyses. The HPLC system was an Agilent Technologies 1260 Infinity II chromatograph coupled to a diode array detector (DAD) with an autosampler. An Eclipse XDB-C18 column (Agilent, 4.6 mm diameter, 250 mm long, and 5 µm size particle) was used to separate the metabolites (Agilent, Santa Clara, CA, USA). The mobile phases were a mix of three solvents, namely, water (A), methanol (B), and phosphoric acid (H_3_PO_4_) 10^−2^ M (C), following the gradient described in Table 6. The column temperature was 30 °C, and the injection volume was 10 µL. The detection of the components was carried out at 230 nm with glucovanillin, vanillin, vanillyl alcohol, *p*-hydroxybenzyl alcohol, and anisyl alcohol; 254 nm for vanillic acid, *p*-hydroxybenzoic acid and anisic acid; and 280 nm for anisaldehyde and *p*-hydroxybenzaldehyde. The components were quantified using an external calibration curve of the 10 standards with a concentration ranging from 50 to 500 ppm. The analysis was performed on each of the 25 cured vanilla pods.

#### 4.6.2. GC-MS Analysis

The extraction was carried out according to the protocol previously established by Pérez-Silva et al. [10]. First, 1 g vanilla pod powder was suspended in 10 mL of distilled water and 80 mL of a pentane ether mix (1:1). The mixture was homogenized in Potter Elvejhem tubes for 2 min. The organic stage was filtered over sodium sulfate anhydrous to eliminate water, and it was concentrated at 1 mL for a Vigreux column at 42 °C, which was to be analyzed using GC-MS. Metabolite separation was carried out using a GC (7890 model) coupled to a mass spectrometer (MSD 5975C) equipped with an HP-5MS capillary column (30 m length, 0.25 mm diameter, and 0.25 µm film thickness), all from Agilent Technologies. The carrier gas was helium (99.99% of purity) at 1.5 mL/min. The injection volume was 1 µL in spitless mode. The oven starting temperature, 40 °C, was maintained for 3 min and increased by 3 °C/min until a final temperature of 250 °C was reached, and maintained for about 5 min. The injector temperature was 220 °C, the detector was 250 °C, and the source temperature was 230 °C. The mass spectra were examined to 70 eV. The components were identified using their retention index and a comparison between the mass spectra based on the library (NIST08 L). The index retention was calculated using an alkene mix (C_8_-C_20_) as a reference. The analyses were performed in triplicate using a mixture of pods of varying sizes.

### 4.7. Fatty Acid Profile Analysis with GC-MS

The fatty acids were determined as methyl ester derivatives (FAMEs) throughout the GC-MS. The FAMEs were prepared by adding 1 mL of KOH 0.5 mol/L to a methanolic solution until it reached the vanilla oily stage (0.05 g), remaining in agitation for 10 min. After saponification, methyl esters were extracted with 1 mL of n-hexane shaking for 5 min; both phases were separated using decantation. The hexane phase was diluted 1/25 (*v*/*v*) before injecting 1 µL in the GC-MS. The chromatographic separation was made using an Agilent fused silica HP-5MS UI capillary column (30 m length, 0.25 mm diameter, and 0.25 μm film thickness). The injector temperature was set to 250 °C; the injection was made in spitless mode, and the gas flow was set at 1 mL/min. The oven temperature was programmed as follows: starting temperature of 100 °C maintained for 1 min, increased by 7 °C/min to 240 °C, and finally increased by 10 °C/min to 325 °C, where it was maintained for 4 min. The mass spectrometer was programmed in just one quadruple in “scan” mode, with the instrumental parameters established at 250, 230, and 180 °C for the transfer line, source, and quadruple, respectively. The collision energy was set at 70 eV, and the data acquisition ranged from *m*/*z* of 5 to 500 with a solvent delay of 2 min. The analyses were performed in triplicate.

### 4.8. Data Analysis

The mean and standard deviation analysis was carried out using the STATISTICA v.10 statistical software package.

## 5. Conclusions

The cured pods of Maya vanilla are physically distinct from commercial vanilla species due to their size and thickness, making them easily identifiable. However, their chemical composition is similar to that of the two most commercially important species (*V. planifolia* and *V. × tahitensis*). The results from the HPLC-DAD and GC-MS analyses indicate that the aromatic profile of *V. cribbiana* Soto Arenas is a combination of the *V. planifolia*, *V. × tahitensis*, and *V. pompona* profiles. The content of vanillyl compounds is higher than in *V. × tahitensis* and *V. pompona*, while the content of anisyl compounds is greater than in *V. planifolia*. Seventy compounds were identified using GC-MS.

Oleic acid was the dominant fatty acid, and together with linoleic acid, these two represented over 82% of the fatty acids in Maya vanilla.

The results of this study demonstrated that the physicochemical and aromatic attributes of Maya vanilla are outstanding and competitive compared to the vanilla species available on the market. This species offers an excellent alternative to mitigate the adverse effects of climate change, promote increased vanilla production, and diversify the vanilla market. It serves as a valuable raw material for producing alcoholic extracts, oleoresins, and other high-value-added products for the food and fragrance industries.

Additionally, this knowledge helps us to understand its domestication, local commercialization, and ethnobotanical use by the Maya culture in southern Mexico and Alta Verapaz, Guatemala, where its production could be expanded.

To further investigate the aromatic profile and bioactive properties of vanilla, the use of alternative extraction techniques and emerging analytical methods will be required.

## Figures and Tables

**Table 1:** Physical characteristics of cured *Vanilla cribbiana* Soto Arenas pods.

Physical Characteristics	Value
Length (cm)	6–16
Weight (g)	2.2–8.2
Humidity (%)	22–38.63

The result ranges are from the measurements of 100 cured vanilla beans.

**Table 2:** Proximate chemical composition of cured *V. cribbiana* Soto Arenas beans.

Component	Content (g/100 g Dry Sample)
Fat	13.96 ± 0.85
Protein	5.07 ± 0.11
Non-soluble fiber	
Lignin	26.42 ± 1.70
Hemicellulose	12.71 ± 0.81
Cellulose	11.06 ± 0.65
Ashes	7.30 ± 0.30
Carbohydrates	23.48 ± 2.10

**Table 3:** Content of aromatic compounds in cured *Vanilla cribbiana* Soto Arenas beans quantified using HPLC-DAD (g/100 g dry matter), Mean ± SD.

Group	TR (min)	Compounds	*Vanilla cribbiana*	*Vanilla planifolia* [37]
	4.10	Glucovanillin	not detected	-
Vanillyl	11.53	Vanillin	2.13 ± 0.68	Minimum 2%
	8.77	Vanillic acid	0.105 ± 0.035	0.0411–0.0861 ppm
	4.60	Vanillyl alcohol	0.055 ± 0.035	-
Benzyl	6.53	*p*-hydroxybenzoic acid	0.380 ± 0.05	0.0058–0.018 ppm
	8.33	*p*-hydrobenzaldehyde	0.345 ± 0.115	0.0219–0.0498 ppm
	3.59	*p*-hydroxybenzyl alcohol	0.085 ± 0.065	-
Anisyl	13.64	Anisyl alcohol	0.065 ± 0.025	-
	19.70	Anisic acid	0.245 ± 0.045	-
	18.58	Anisaldehyde	not detected	-

**Table 4:** Volatile compounds identified with GC-MS in cured *Vanilla cribbiana* Soto Arenas beans.

Volatile Compounds	IR Exp. (Lit.) *	Aromatic Descriptors **
Acids (14)		
Acetic acid	643 (623)	Vinegar
Benzoic acid	1177 (1193)	Wine, very weak, and balsamic
Nonanoic acid	1275 (1297)	Green, fatty, moldy, bitter, oily, caprylic, and cheesy
Benzoic acid, 4-methoxy- (*p*-anisic acid)	1448 (1451)	Sweet
Benzoic acid, 4-hydroxy- (*p*-hydroxybenzoic acid)	1535 (1558)	
Dodecanoic acid (lauric acid)	1585 (1570)	Dry, metallic, weak, fatty, and waxy
Benzoic acid, 4-hydroxy-3-methoxy- (vanillic acid)	1600 (1608)	Dairy, sweet, creamy, and vanilla
Tetradecanoic acid (myristic acid)	1770 (1765)	Very weak, waxy, and oily
Pentadecanoic acid	1868 (1869)	Waxy
Hexadecanoic acid (palmitic acid)	1974 (1964)	Oily
Octadecanoic acid (stearic acid)	1996	Fatty
9-octadecanoic acid (oleic acid)	2095 (2082)	Fatty
9,12-octadecadienoic acid (linoleic acid)	2125 (2130)	Faint, fatty
Cis-13-octadecenoic acid	2280	
Alcohols (14)		
2,3-butanediol	766 (769)	Fruit
Phenol	987 (981)	Phenolic and medicinal
1-hexanol, 2-ethyl-	1032 (1027)	Rose and green
Benzyl alcohol	1039 (1035)	Aromatic, flower, and fruit
1-octanol	1074 (1073)	Herbal, fatty, and green
4-methyl phenol, (*p*-cresol)	1080 (1098)	Smoked and phenolic
2-methoxy-phenol, (*o*-guaiacol)	1091 (1090)	Smoked, sweet, phenolic, chemical, and medicinal
Bicyclo [2.2.1]heptan-2-ol, 1,3,3-trimethyl- (fenchol)	1114 (1121)	Camphor, earthy, pine, citric, and wood
Phenylethyl alcohol	1117 (1117)	Honey, sweet, yeast, floral, spicy, herbal, rose, and lilac
Borneol	1168 (1165)	Camphor and pine
2-methoxy-4-methyl phenol (*p*-creosol)	1199 (1199)	Spices, vanilla, phenolic, and medicinal
4-methoxybenzyl methanol (*p*-anisyl alcohol)	1287 (1273)	Sweet, flower, and anise-like
2-methoxy-4-vinylphenol (*p*-vinylguaiacol)	1315 (1315)	Clove, phenolic, and smoked
4-hydroxy benzenemethanol (*p*-hydroxybenzyl alcohol)	1396 (1357)	Almond, sweet, and coconut
Aldehydes (8)		
Octanal	1003 (1005)	Citric, herbal, flower, fruit, and orange
Nonanal	1101 (1104)	Fat, citrus, and green
4-methoxybenzaldehyde (*p*-anisaldehyde)	1256 (1263)	Mint, sweet, and anise-like
*p*-hydroxybenzaldehyde	1406 (1391)	Sweet, nutty, almond, balsamic, woody, vanilla-like, and biscuit
4-hydroxy-3-methoxybenzaldehyde (vanillin)	1416 (1406)	Vanilla and sweet
Benzaldehyde, 3,4-dihydroxy-(protocatechualdehyde)	1605	Almond and peach
Benzaldehyde, 4-hydroxy-3,5-dimethoxy-(syringaldehyde)	1668 (1662)	Green and herbal
4-methoxy-3-phenoxymethylbenzaldehyde	1871	
Esters (14)		
Acetic acid, 2-ethylhexyl ester (octyl acetate)	1154	Sour and vinegar-like
Benzyl acetate	1171 (1165)	Flower, fruit, and pear
Benzoic acid, 4-methoxy-, methyl ester (methyl *p*-anisate)	1377 (1376)	Sweet, herbal, and flower
2-propenoic acid, 3-phenyl-, methyl ester (methyl cinnamate)	1385 (1388)	Fruit
Benzoic acid, octyl ester (2-octyl benzoate)	1712	
Myristic acid, methyl ester (methyl tetradecanoate)	1727 (1725)	Waxy and cognac
1,4-benzenediol, 2,5-bis(1,1-dimethylethyl)-	1730	
Tetradecanoic acid, 2-methyl-, methyl ester	1796	
Hexadecanoic acid, methyl ester	1927 (1926)	
Hexadecanoic acid, ethyl ester	1996 (1993)	
11-octadecenoic acid, methyl ester	2051	
Dodecanoic acid, phenylmethyl ester (benzyl laurate)	2337	
Octadecanoic acid, 2-methylpropyl ester	2220	
Octadecanoic acid, phenylmethyl ester (benzyl stearate)	2502	Fruit and pear
Furans (2)		
2,3-dihydrobenzofuran (coumaran)	1222 (1219)	Sweet and cinnamon
5-hydroxymethylfurfural	1240 (1233.2)	Tobacco, hay, herbal, chamomile tea, butter, and caramel
Hydrocarbons (12)		
Dodecane	1200 (1200)	
Thiophene, 2,5-bis(2-methylpropyl)	1514 (1533)	Sulfur and garlic
Hexadecane, 4-methyl-	1659	
Cyclotetradecane	1679 (1673)	
Heptadecane	1700 (1700)	
Pentadecane, 2,6,10,14-tetramethyl (norphytan)	1706 (1707)	
Hexadecane, 7,9-dimethyl-	1743	
1-octadecene	1764 (1795)	
Octadecane	1799 (1800)	Sweet and fruit
Hexadecane, 2,6,10,14-tetramethyl-	1809 (1811)	
Nonadecane	1899 (1900)	
1,1,4β,7-tetramethyl-, [4aS-(4α,4β,7α,8α)]-(rimuen)	1911 (1894)	
Ketones (6)		
3-hydroxybutan-2-one (aceotoin)	709 (718)	Fatty, cream, and buttery
2-cyclohexen-1-one, 2-methyl-	1192	
1-butanone, 1-(2,4,5-trihydroxyphenyl)-	1498	
*p*-hydroxybenzalacetone	1740	
Dehydrozingerone	1823 (1822)	Vanilla, creamy, and balsamic
7,9-di-tert-butyl-1-oxaspiro(4,5)deca-6,9-diene-2,8-dione	1922 (1929)	Sulfur
Total number of identified compounds	70	

* Kovats IR: Retention index in an HP-5MS capillary column using alkane mix C_8_–C_20_, Exp: experimental; Lit: literature (https://webbook.nist.gov/chemistry/name-ser/; https://www.pherobase.com, accessed on 1 July 2024), Pérez-Silva et al. [10]. ** aromatic descriptors in the literature (https://www.pherobase.com/; https://www.flavornet.org, accesse on 1 July 2024), Pérez-Silva et al. [10] and Zhang and Mueller [12].

**Table 5:** Fatty acid profile in cured vanilla beans of *V. cribbiana* Soto Arenas (Maya vanilla).

Fatty Acids	Relative Percentage (%)
Dodecanoic acid (lauric acid)	0.61
Tetradecanoic acid (myristic acid)	3.57
Hexadecanoic acid (palmitic acid)	11.48
Octadecanoic acid (stearic acid)	1.33
Saturated	16.99
9-octadecanoic acid (oleic acid)	52.98
11-eicosenoic acid (gondoic acid)	0.58
15-tertacosenoic acid (nervonic acid)	0.05
Monounsaturated	53.61
9,12-octadecadienoic acid (linoleic acid)	29.41
Polyunsaturated	29.41

**Table 6:** Gradient used for elucidation of the compounds with HPLC-DAD.

t (min)	%A	%B	%C	Flow (mL/min)
0	71	19	10	1.5
3	71	19	10	2.0
9	71	19	10	2.0
15	55	35	10	2.0
18	51	39	10	2.0
21	48	42	10	2.0

## Data Availability

The original contributions presented in the study are included in the article/Supplementary Material; further inquiries can be directed to the corresponding author.

## Supplementary Materials

The following supporting information can be downloaded at: https://www.mdpi.com/article/10.3390/plants14030300/s1, Figure S1. Harvest of fruits *Vanilla cribbiana* Soto Arenas (Maya vanilla) from “Rápidos Che’ sib’ ik” Farm, Coban, Alta Verapaz, Guatemala. Photographs by Darío Ramírez-Fontana (2024). Figure S2. Start of the curing process *Vanilla cribbiana* Soto Arenas beans (Maya vanilla) from “Rápidos Che’ sib’ ik” Farm, Coban, Alta Verapaz, Guatemala. Photographs by Darío Ramírez-Fontana (2025).
