# Supplementary material for: Maya Vanilla (Vanilla cribbiana Soto Arenas): A New Species in Commerce
Source: Plants (Basel). 2025 Jan 21;14(3):300. doi: 10.3390/plants14030300 (PMC11820238; doi:10.3390/plants14030300)
Supplement: Supplementary file 1 [file plants-14-00300-s001.zip › plants-3372975-supplementary.pptx]

## Slide 1
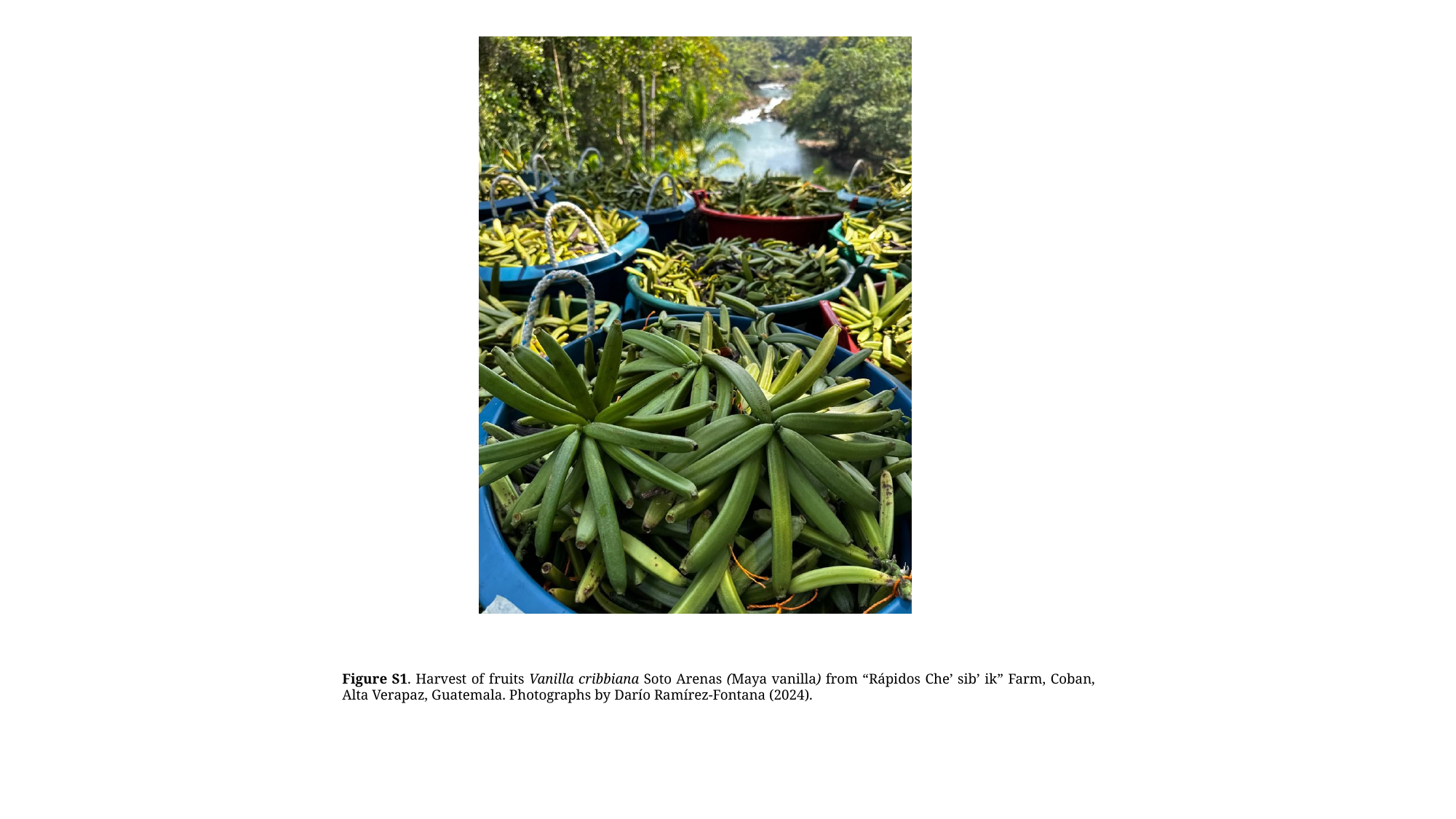

Figure S1. Harvest of fruits Vanilla cribbiana Soto Arenas (Maya vanilla) from “Rápidos Che’ sib’ ik” Farm, Coban, Alta Verapaz, Guatemala. Photographs by Darío Ramírez-Fontana (2024).

## Slide 2
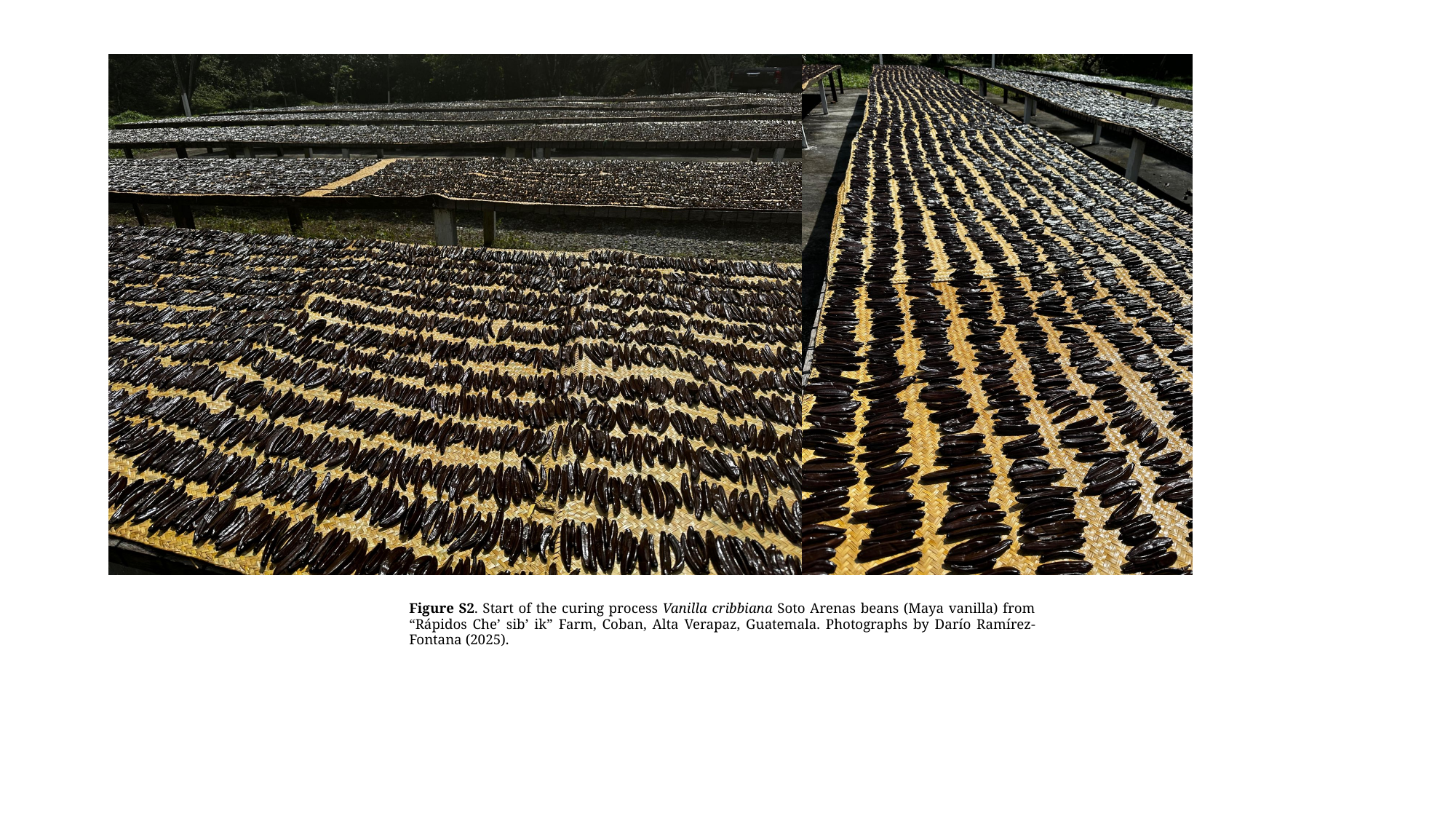

Figure S2. Start of the curing process Vanilla cribbiana Soto Arenas beans (Maya vanilla) from “Rápidos Che’ sib’ ik” Farm, Coban, Alta Verapaz, Guatemala. Photographs by Darío Ramírez-Fontana (2025).
